# Antineutrophil Cytoplasmic Antibody (ANCA) Vasculitis, Fatigue, and Treatment With Avacopan: A Case Series

**DOI:** 10.7759/cureus.94987

**Published:** 2025-10-20

**Authors:** Carl Schulze

**Affiliations:** 1 Nephrology, University of California Los Angeles, Los Angeles, USA

**Keywords:** anca-associated vasculitis, avacopan, chronic fatigue, complement inhibitor, mpo-anca, pauci-immune crescentic glomerulonephritis, small vessel vasculitis

## Abstract

This report describes three patients who developed marked somnolence and fatigue during treatment with avacopan for antineutrophil cytoplasmic antibody (ANCA) vasculitis. Though fatigue is a common symptom in ANCA vasculitis, each of these patients reported noticeable worsening of fatigue after starting avacopan, with improvement shortly after stopping the medication. All of them had a good response to treatment of their underlying vasculitis, and none of them had other clear causes of worsening fatigue.

## Introduction

Physicians have historically defined successful treatment in terms of reducing mortality and improving other objective markers of disease. Modern treatment of antineutrophil cytoplasmic antibody (ANCA) vasculitis has achieved this aim, dramatically reducing the rate of mortality and organ failure [1]. The vast majority of ANCA vasculitis cases have now become chronic, relapsing-remitting diseases, but a major ongoing problem is that of patient fatigue [2]. Approved on 10/07/2021 for the treatment of adults with severe ANCA vasculitis as an adjunct with prednisone, avacopan is a recent addition to the toolkit in treating ANCA disease [3]. The preclinical data suggested a positive effect of avacopan on health-related quality of life (HRQOL). The initial phase 2 "clear" trial showed improvement in the physical and mental functioning of the Short-Form 36 Health Survey (SF-36) and the EuroQol-5D-5L (EQ-5D-5L) tool [4], and the second "classic" trial showed improvement in SF-36 domains of pain, vitality, mental health, physical functioning, and emotional role [5]. The ADVOCATE trial similarly showed improvement in HRQOL on SF36 and EQ-5D-5L scales [6] in the avacopan arm. The only published data on fatigue with avacopan come from the package insert, which reports it to be slightly more common with avacopan (10.2%) as compared with placebo (9.1%) [7]. What follows is a report of three patients who experienced increased fatigue and somnolence after starting avacopan, one of whom led to premature cessation of therapy. 

## Case presentation

Case 1

This is the case of a 48-year-old female patient with relapsed ANCA vasculitis. Her disease has mostly been limited to the kidneys, and the myeloperoxidase (MPO) antibody was weakly positive once, but otherwise the ANCA testing has always been negative. She had been in remission for several years before developing a relapse characterized by foamy urine, increased creatinine, and increased proteinuria. A kidney biopsy showed focal active ANCA-associated glomerulonephritis and mesangial immunoglobulin-A (IGA) deposits, focal glomerulosclerosis, and moderate interstitial fibrosis/tubular atrophy.

Induction treatment was pulse methylprednisolone and rituximab (375 milligrams per square meter of body surface area (mg/m^2^) x four doses). She did not get an oral steroid taper due to severe mental side effects of pulse steroids. Avacopan 30 mg twice a day was started around the time of completion of rituximab. About five months later, she developed prominent fatigue and reported increased somnolence. There was no evidence for relapse of vasculitis. Metabolic panels were normal, including sodium, calcium, and transaminases. Complete blood count showed mild anemia (hemoglobin 10.5-12 grams per deciliter (g/dL)). Thyroid function testing was normal. 

The avacopan was not initially stopped, as the fatigue seemed to start after another medication (dapagliflozin) was started. However, after the avacopan was stopped at 12 months (the standard course of treatment), she felt noticeably better within a few weeks. She remains on dapagliflozin to slow the progression of proteinuric chronic kidney disease and has not experienced fatigue or somnolence over the past six months. There is otherwise no evidence for vasculitis activity, with resolution of hematuria and improvement in proteinuria.

Case 2

This is the case of a 68-year-old male patient who developed MPO-positive ANCA vasculitis with accelerated chronic kidney disease, proteinuria, microhematuria, and interstitial lung disease. He was treated with pulse methylprednisolone, rituximab (375 mg/m2 x 4 doses), and oral prednisone taper. Avacopan 30 mg twice daily was started about two weeks after completion of rituximab, which was during the oral prednisone taper (delay was due to unclear ability of the patient to follow up due to his travel schedule).

He developed hypersomnolence, which lasted about five hours after each dose of avacopan, and he self-discontinued the medication within three weeks. The somnolence did not recur after stopping avacopan. The renal vasculitis remains in remission, with creatinine improved, and resolution of hematuria and proteinuria. However, his lung disease became somewhat worse about six months after being weaned off prednisone, requiring reintroduction of prednisone. 

Case 3

This is the case of an 82-year-old female patient with MPO-positive ANCA acute glomerulonephritis who was also treated with methylprednisolone, rituximab, oral prednisone taper, and avacopan. She developed worsening fatigue and somnolence six weeks after starting avacopan and at one follow-up visit reported feeling “like a zombie.” She had preexisting health conditions which likely contributed to fatigue, notably idiopathic pulmonary fibrosis, but after starting avacopan, fatigue and somnolence because the most prominent symptoms. 

About six months into the treatment course, the avacopan was held due to fatigue, and an elevated alanine aminotransferase (ALT) was observed, so she was advised to continue to hold avacopan along with other medications, possibly causing the transaminitis. Her fatigue did initially improve, though it persisted with some waxing and waning. She eventually resumed the medication after the ALT had normalized and completed a 12-month course. Since coming off avacopan, she has been maintained on rituximab and nintedanib for interstitial lung disease and has reported milder fatigue. The vasculitis remains in clinical remission, with improvement in kidney function, elimination of hematuria, and normalization of proteinuria. The MPO remains positive but at a lower level than before treatment.

A summary of the patients' clinical changes is presented in Table 1. 

**Table 1:** Select patient characteristics MPO: myeloperoxidase; MP: methylprednisolone; GC: glucocorticoids; CKD: chronic kidney disease; Cr: creatinine; mg/dL: milligrams per deciliter; ILD: interstitial lung disease

Case	1	2	3
Demographics	48 yo white female	68 yo Asian male	82 year old white female
Relapse or de novo	Relapse	De novo	De novo
Duration	>10 years	2 years	1 year
ANCA subtype	MPO	MPO	MPO
Organ Involvement	Kidneys only	Kidneys, lungs	Kidneys, preexisting lungs
Treatment	Rituximab, avacopan	Rituximab, pulse MP, GC taper	Rituximab, pulse MP, GC taper
Onset of fatigue	5 mo after avacopan	1 week after avacopan	6 weeks after avacopan
Outcome of fatigue	Improved after stopping avacopan	Resolved after stopping avacopan	Partial improvement after stopping avacopan
Renal outcome	Stable CKD, resolved hematuria, proteinuria reduced by 75%	Improvement in Cr from 2.7 to 1.5 (mg/dL); resolved proteinuria and hematuria	Improvement in Cr from 2.3 to 1.4 (mg/dL); resolved proteinuria and hematuria
Comorbid conditions	Anemia of CKD	Flare of ILD 1 year later	Waxing and waning ILD symptoms
Current ANCA	Negative	Positive	Positive

## Discussion

ANCA vasculitis has become a chronic condition with a substantial burden of comorbid conditions, reduced quality of life, and fatigue [8]. Fatigue occurs in about 75% of patients and is associated with anxiety, depression, pain, and sleep disturbances [8]. It is the symptom of greatest concern to patients and leads to reduced quality of life, reduced functioning in basic daily tasks, increased social stigma, and interference with work [9-12]. Myalgic encephalomyelitis/chronic fatigue syndrome (ME/CFS) shares many similarities with fatigue associated with ANCA vasculitis, and a cross-sectional study in Canada recently found that 52% of patients with ANCA vasculitis met diagnostic criteria for ME/CFS [13]. 

Fatigue in ANCA vasculitis leads to worse perceptions of physical health than those of mental health [14]. A study looking at cardiopulmonary fitness found no difference between patients with ANCA vasculitis and controls, leading to the conclusion that the fatigue was central in nature [15]. Disease duration also does not matter, with similar amounts of fatigue reported across variable durations of disease [14,16]. Treatment of underlying vasculitis leads to incomplete resolution of fatigue [9, 17]. Some studies show more severe fatigue in patients with MPO versus proteinase-3 (PR3) and microscopic polyangiitis (MPA) versus granulomatosis with polyangiitis (GPA) clinical patterns, though this is not universal [16].

Information about the mechanisms of fatigue in vasculitis could offer clues about what could have happened with the patients above. Multiple mechanisms have been implicated in fatigue, including oxygen and nutrient supply, metabolism, daytime sleep, mental health, and inflammation [11]. Central nervous system (CNS) inflammation might be a particularly important factor in causing fatigue, as other non-autoimmune conditions affecting the CNS, including malignancy, stroke, and traumatic brain injury, are also associated with fatigue [11]. Many cell signaling molecules are implicated in fatigue, including interleukin 1-beta (IL-1β), tumor necrosis factor alpha (TNF-α), and interferon gamma (IFN-γ) [11]. Patients with ME/CFS and fatigue in ANCA vasculitis have similarities in mitochondrial dysfunction, perturbations in T-helper cell 2 (Th2) polarization, low immunoglobulin G (IgG) levels, and reduced natural killer (NK) cell cytotoxicity [16]. Activation of complement has also been shown to cause fatigue in ME/CFS [17] and paroxysmal nocturnal hemoglobinuria [18]. 

Treatment of vasculitis, which targets the inflammatory cause of the disease, does not always improve fatigue [10]. A cohort study using the SF-36 from two clinical trials for ANCA vasculitis found that many patients had improvement in fatigue (the vitality domain of the SF-36) over the first six months of treatment, but still had higher fatigue than controls, and a subgroup of patients did not improve at all [9]. The patients without improvement in fatigue had lower vasculitis activity, but no differences in kidney function, C-reactive protein levels, or other evidence for tissue damage, as compared to patients with improvement in fatigue [9]. A post hoc study of the ADVOCATE trial showed some benefit to SF-36 scores and other markers of quality of life during treatment with avacopan, although the vitality portion (most reflective of fatigue) of the SF-36 did not improve [19]. 

The patients above experienced the opposite of what was hoped, namely, that their fatigue worsened after treatment. It is unclear how complement inhibition, and avacopan in particular, might affect fatigue. Avacopan is a small molecule inhibitor of the receptor to complement 5a (C5a), which leads to reduced activation and chemoattraction of neutrophils [6]. Other complement inhibitors, the C5 inhibitors eculizumab and ravilizumab, also had increased rates of fatigue in their package inserts (approximately 5-10%) [20,21], though the mechanism is unclear, and these medications were shown to improve fatigue in other conditions [18,22]. Steroid withdrawal could possibly cause fatigue to worsen, though only the second and third patient cases described above received an oral steroid taper, and an accelerated steroid taper was not associated with worse quality of life in the PEXIVAS trial in ANCA vasculitis [23]. 

Indeed, fatigue in ANCA vasculitis is very common in all groups of patients. The mechanisms of fatigue have been partly elucidated, but many questions remain, especially whether therapeutic targets exist to treat fatigue directly. There are currently no treatments for fatigue in ANCA vasculitis, though a regimen of transcranial direct current stimulation combined with low-intensity aerobic exercise recently showed benefit in two patients [24].

## Conclusions

The above patients had marked worsening of fatigue after starting avacopan and improvement after stopping it. They all reported fatigue and somnolence as prominent symptoms, and formal scales like the SF-36 were not collected, which admittedly reduces the precision of monitoring the evolution of their symptoms. The timing is striking, but there is no reported mechanism for how this might have happened. The literature on complement inhibition is somewhat conflicting, with some groups having reduced rates of fatigue but others having increased rates. All of the patients above had MPO-positive ANCA vasculitis, where fatigue is especially common. All of them had comorbid conditions, which could have been contributing to fatigue as well. Nonetheless, it will be noteworthy to see if any other reports of fatigue emerge associated with avacopan or similar medications, as well as clues about potential mechanisms.
